# A dynamic management model for sustainable drug supply chain in hospital pharmacies in Iran

**DOI:** 10.1186/s12913-024-11692-8

**Published:** 2024-10-09

**Authors:** Elham Shahbahrami, Rohollah Kalhor, Mohammad Amerzadeh, Mahdi Hasani, Mehrdad Kiani

**Affiliations:** 1 Health Management and Economics Research Center, Iran University of Medical Sciences, Tehran, Iran; 2 Non-communicable Diseases Research Center, Research Institute for Prevention of Non-Communicable Diseases, Qazvin University of Medical Sciences, Qazvin, Iran; 3 Research Center for Cognitive & Behavioral Sciences in Police, Directorate of Health, Rescue & Treatment, Police Force, Tehran, Iran; 4 Faculty of Economics, Management and Accounting, Department of Industrial Management, Yazd University, Yazd, Iran

**Keywords:** Medicine, Pharmacy, System dynamics, Sustainability, Supply chain management

## Abstract

**Background:**

Sustainable supply chain management encompasses the strategic coordination and control of material, information, and financial flows, as well as the collaborative efforts among the entities engaged in the medicinal supply chain. This research proposes a dynamic and sustainable supply chain management model tailored explicitly for the inpatient pharmacies of Medical Centers and Hospitals affiliated with Iran University of Medical Sciences.

**Methods:**

This is a quantitative study in terms of research objective and a qualitative study based on the stages in the conceptual development of the model. Therefore, the current study can be considered a mixed-methods approach. After identifying the key factors influencing the sustainability of the medicine supply chain, we conducted a dynamic analysis of the problem using system dynamics methodology. In order to simulate the system’s behavior over 24 months, we utilized a combination of existing documentary information and expert opinions. The developed model was implemented using Vensim PLE software, allowing us to simulate and analyze the impact of various policies on the system.

**Results:**

Medicine disposal exhibited an upward trend, particularly during the second 12-month period. Conversely, the trend of medicine expirations remained relatively stable in the initial months but showed an upward trajectory after that. The cost associated with disposed medicine experienced a consistent increase, with a higher rate observed during the second 12-month period. In contrast, sales of low-consumable medicine experienced a significant initial surge followed by a slower growth rate. Finally, the pharmacy’s profit demonstrated an overall increasing trend, although the rate of increase was higher during the first 12 months.

**Conclusion:**

Among the various scenarios considered, namely “increasing the adequacy of human resources,” “increasing the speed of response,” and “utilizing pharmacists in the drug prescribing team,” it was found that these interventions had a substantial effect on both enhancing the pharmacy’s profit and reducing medication waste. Therefore, these scenarios were identified as having the most significant impact. The proposed model can serve as a valuable tool for forecasting and informing policy-making, providing insights into addressing the challenges associated with the sustainable drug supply chain in hospital pharmacies.

## Background

The growing awareness among the general public regarding social and environmental issues has generated significant interest among researchers and experts in sustainability concepts during the past two decades [1]. As a result, the concept of sustainable supply chain management has emerged as a focal point within management studies. Sustainable supply chain management encompasses the effective management of material, information, and capital flows and fostering collaboration among the entities involved in the medicine supply chain. Furthermore, it addresses all three dimensions of sustainability, namely economic, environmental, and social aspects [2].

Sustainable supply chain management comprises four primary dimensions: sustainable product design, efficient process flows, and sustainable collaboration with suppliers and customers [3]. For researchers, the sustainable development of supply chain management is considered a constraining factor and an approach for enhancing performance, competitiveness, and overall supply chain organization [4]. This approach facilitates better cost management, improved operational performance, enhanced product quality, and the creation of a competitive advantage for organizations [5]. However, it is crucial to note that improper implementation of supply chain management can lead to significant investment losses for the organization [6].

Effective management of the medicine supply chain is paramount for the healthcare system. The medicine supply chain possesses unique characteristics that differentiate it from other goods. These include high costs, lengthy research and development processes, short product life cycles, governmental regulations, reduced patent periods, stringent production regulations, and storage, distribution, and consumption complexities. Consequently, the medicine supply chain is highly intricate, and its management presents significant sensitivity and challenges. Therefore, maintaining a customer service level below 100% is unacceptable [7].

In Iran, the distribution of pharmaceuticals is currently encountering numerous challenges, as reported by officials. These challenges have resulted in medication shortages and an escalation in the quantity of expired medications. Consequently, individuals have often difficulty accessing the necessary medications to meet their healthcare needs [8]. The provision of medications is recognized as the eighth crucial component of primary healthcare, and it constitutes one of the essential responsibilities of the government. Additionally, the pharmacy plays a significant role in delivering medical services to patients, both in outpatient and inpatient settings [9].

Given the pharmaceutical industry’s significant global importance, researching to enhance community health and foster competitiveness is integral to a country’s strategic studies [10]. Fortune Magazine, in its annual selection of the most profitable industries, has consistently recognized the pharmaceutical industry as the top performer for multiple years. This industry’s significance, coupled with the existing research gaps within various aspects of the medical supply chain, underscores the necessity of studying and conducting research in this field [11].

Furthermore, the sustainable medical supply chain faces distinct challenges across all its products and activities, and these challenges, along with their underlying causes, can disrupt the seamless functioning of the sustainable supply chain. Therefore, effectively managing and mitigating the complexity of these challenges within the sustainable supply chain is paramount [12].

Considering the significance of sustainability and the adverse impact of industries on it, sustainable supply chain management emerges as an approach that seeks to improve processes and products in alignment with economic, social, and environmental requirements. Consequently, analyzing the factors influencing the sustainability of the supply chain and studying the linear and nonlinear relationships between variables become crucial steps towards enhancing its management. In this regard, the systems dynamics approach proves valuable in understanding the dynamics and effectiveness of these factors, thereby proposing a principled and efficient model to address the problem. Consequently, this study aims to present a dynamic and sustainable supply chain management model specifically tailored for the pharmacy sector in educational hospitals.

## Methods

The study can be categorized as a mixed-methods approach. Mixed methods are ‘research in which the investigator collects and analyses data, integrates the findings, and draws inferences using qualitative and quantitative approaches or methods in a single study’ [13]. In the qualitative part, using the literature review, the researchers examined previous research and theories related to the topic. This work helps to identify key and main variables that should be included in the conceptual model. A literature review can also help identify gaps in current knowledge and determine the need for further research. After identifying the variables, qualitative data was collected by interviewing experts. The information obtained from these interviews helped to develop and enrich the conceptual model.

In the quantitative part, system dynamics was used to test and evaluate the model. The variables used in this study are from an article published in the Journal of Health Management [14]. The information required to perform the study and the simulation includes the initial values of the surface variables and some auxiliary variables at the beginning of the simulation period. We collected the information for the simulation model from technical managers and pharmacists of 16 inpatient pharmacies of medical centers and hospitals in Iran University of Medical Sciences. Interviews were conducted with technical managers and pharmacists from 16 inpatient pharmacies of medical centers and hospitals affiliated with Iran University of Medical Sciences to collect information for the simulation model. These purposefully identified interviews with experts provided qualitative insights and input to inform the development of the state-flow model. In the formulation and testing stages of the model, the researchers relied on a review of existing documents to gather data.

The study, spanning 24 months, indicates a substantial timeframe dedicated to understanding and analyzing the complex and dynamic nature of medicine supply chain management. The researchers employed the system dynamics approach to address this complexity, utilizing Vensim PLE Software.

System dynamics is a methodology that aids in comprehending intricate systems, forecasting trends over time, and designing effective control policies. It provides insights into the dynamic behavior of a system by revealing causal feedback loops that illustrate the relationships among various system components, stock and flow structures, time delays, and nonlinear effects. By employing system dynamics, researchers can better understand the interdependencies and feedback mechanisms within the medicine supply chain and develop strategies to manage it more effectively [15].

The primary objective of system dynamics is to facilitate managers’ comprehension of intricate systems and enable their intervention to ensure that the system’s behavior aligns with their intended objectives. System dynamics entails modeling and simulating complex physical and social systems, thereby facilitating the testing of models and formulating strategies for management and change [15]. System dynamics modeling is a potent tool for comprehending and leveraging the interplay among factors within complex management systems. These models offer an operational framework supporting business planning and decision-making processes [16]. Consequently, system dynamics modeling proves beneficial in scenarios involving the understanding of how strategies unfold over time, the identification of potential error sources, and the determination of measures that can be implemented to mitigate such occurrences [17].

The system dynamics approach finds its principal application in situations where the behavior of a phenomenon is influenced by the inherent dynamics and interactions of the system’s endogenous variables. The system dynamics method can effectively analyze the system when undergoing its typical and natural progression. This approach is built upon the fundamental assumption that complex real-world conditions can be explained by identifying factors and understanding the interconnected relationships among these factors. The primary focus lies in constructing a structural framework that captures the interactive relationships between factors, ultimately giving rise to dynamic behavior within the system [18].

According to Sterman, the creation of a system dynamics model involves five steps:

1. Problem Articulation: This step involves defining the purpose and objectives of the modeling exercise. It determines the specific problem or issue that the model aims to address.
2. Formulation of the Dynamic Hypothesis: A conceptual representation of the complex system is developed in this step. It involves identifying the key variables, their relationships, and the feedback loops that drive the system’s behavior.
3. Formulation of a Simulation Model: The conceptual representation is translated into a simulation model. Equations describe the relationships and interactions among the variables, enabling the model to simulate the system’s dynamic behavior over time.
4. Model Testing: This step involves calibrating the model and verifying its performance. Partial and full-model simulations are compared to available data and the expectations of model users. Various tests are conducted, such as the Structure Verification Test and the Boundary-Adequacy (Structure) Test. These tests assess whether the model’s structure aligns with existing knowledge about the natural system, includes all relevant structures and variables, and satisfies the study’s goals.

It is important to note that while sensitivity analysis is a valuable test for examining the robustness of the model, it was not performed in the present study due to the limitations of the Vensim PLE software and the absence of sensitivity analysis runs. The Structure Verification Test evaluates whether the model’s structure accurately represents the known structures of the actual system. The Boundary-Adequacy (Structure) Test assesses whether the model includes all the necessary structural relationships and variables to address the research goals effectively.

The Structure Verification Test in system dynamics modeling involves comparing the model’s parameters with existing knowledge about the actual system. This test aims to confirm the parameters in terms of their definition and selected values. It raises questions about whether the parameters conceptually and numerically align with the real-world system.

During the Structure Verification Test, considerations include whether the parameters are meaningful and representative of real-world entities or phenomena. It examines whether the selected parameter values are detectable and observable in the natural system or if they are merely introduced to achieve balance within the model’s equations. Additionally, the test assesses whether the chosen parameter values are consistent with available information and data about the actual system.

The fifth step in system dynamics modeling, Policy Design, and Evaluation, involves leveraging scenarios by modifying parameters or structures within the model to examine the impacts of change. This step focuses on designing and evaluating policies or interventions to improve the system’s behavior or achieve desired outcomes [15].

## Results

### Step 1. Problem articulation

The main problem addressed in this research is focused on sustainable drug supply chain management within inpatient pharmacies. Specifically, the study aims to investigate the dynamic and complex interactions among various factors to determine the policies that can enhance profitability and reduce the amount of disposed medicines.

### Step 2. Formulation of the dynamic hypothesis

This study formulated dynamic hypotheses based on the literature review and expert opinions. Seventeen challenges that can impact the efficiency and effectiveness of sustainable drug supply chain management in pharmacies were identified, and their relationships were examined using the fuzzy Delphi method. We defined the dynamic hypothesis as follows: Insufficient specialized human resources, poor responsiveness in emergencies, unnecessary prescription of drugs by residents, weaknesses in financial analysis, and expiration of certain medications are significant challenges in managing sustainable drug supply chains. Addressing and mitigating these challenges can improve financial issues and profitability for pharmacies.

### Step 3. Formulation of a simulation model

At this research stage, two critical diagrams are designed: the cause and effect diagram and the stock and flow diagram. These diagrams serve as visual tools to represent and explain the relationships and dynamics within the system being studied.

### Design a cause-and-effect diagram

**Fig. 1:**
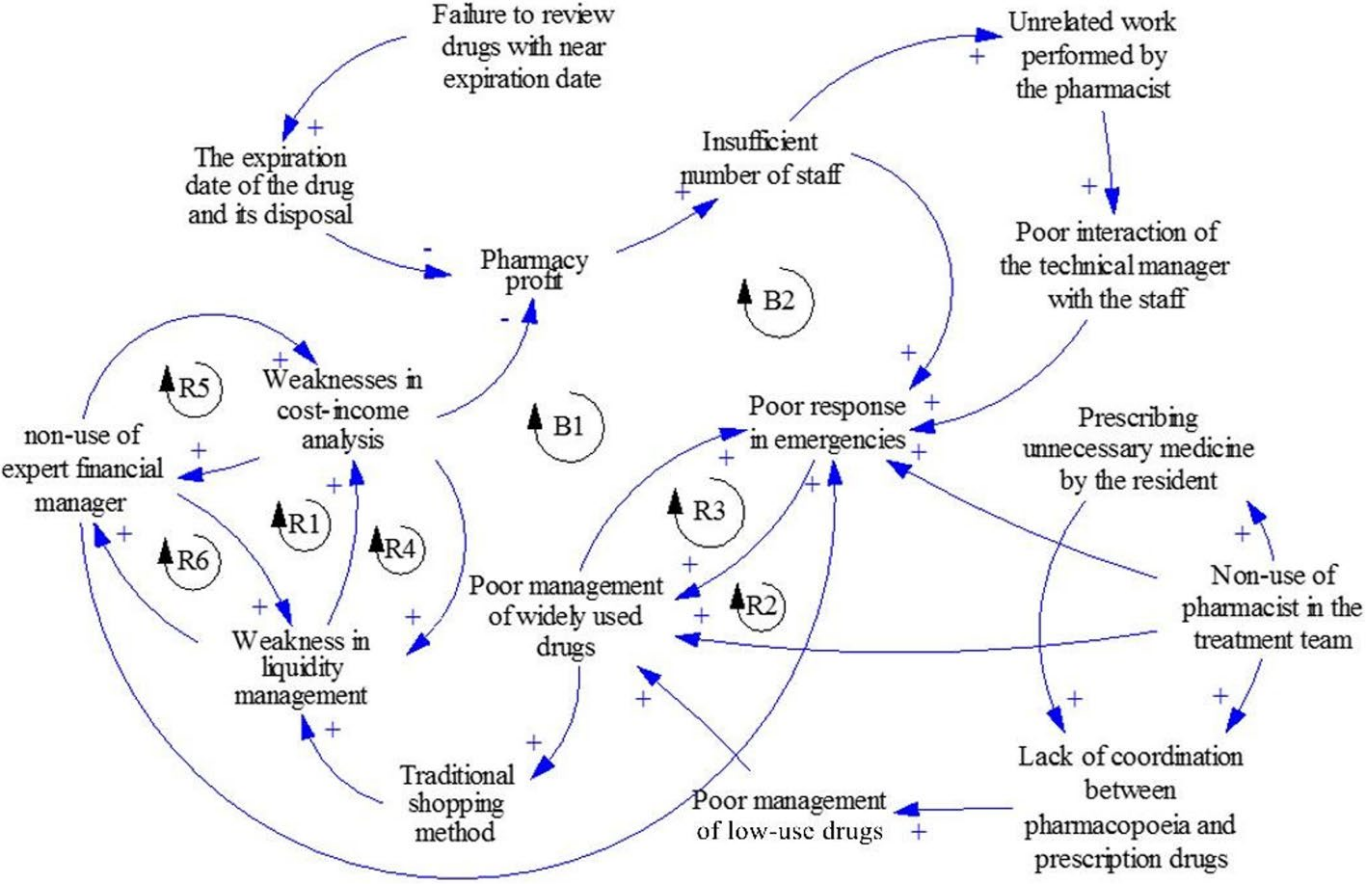
Causal diagram of sustainable drug supply chain management

We utilized the fuzzy Delphi method to identify the causal relationships among the 17 identified challenges. The results indicated that ten challenges were of the causal type, while seven were of the effect type, forming eight loops. For example, two loops, B1 and R1, are described. Figure 1 illustrates the cause-and-effect diagram of sustainable drug supply chain management.

In loop B1, poor emergency responsiveness leads to incorrect prioritization in drug procurement. Consequently, frequently used drugs that require immediate supply may be procured at the last moment. This, in turn, creates difficulties in managing these drugs and prevents a thorough analysis of drug consumption patterns in the pharmacy, hindering accurate prediction of future needs and causing problems in purchasing and maintaining appropriate drug inventory. Furthermore, this issue negatively impacts financial aspects such as liquidity, cost analysis, and pharmacy profitability. Since financial matters are among the most critical factors in attracting personnel to any organization, including pharmacies, it also hurts the sufficiency and recruitment of personnel.

As depicted in the R1 loop, the lack of a certified financial manager in the pharmacy results in inadequate liquidity management, which hampers the pharmacy’s cost-profitability analysis. Similarly, the insufficient analysis of the cost-benefit balance contributes to the decision not to employ a qualified financial manager.

### Stock and flow diagram

This diagram illustrates the interrelationships among variables within a system and serves as a fundamental framework for constructing a quantitative model. Comprehending two fundamental concepts, namely stock and flow, is crucial when creating a Stock and Flow Diagram. Stock variables represent the state or condition of the system at a specific point in time. Even when considering time, these variables can still be defined. Examples of stock variables include the inventory of products or the number of personnel within a company. These variables provide a snapshot of the system’s status at a given moment and can change over time due to various flows occurring within the system.

In contrast, flow variables are contingent upon time and cannot be defined independently of it. They capture the rates of change or movement between stocks. Flow variables are influenced and modified by stock variables. For instance, when an order is dispatched, the stock of products is reduced. Similarly, the production of a product results in an increase in the stock of products. Other examples of flow variables encompass sales, employee turnover, or inflow and outflow rates of financial resources.

**Fig. 2:**
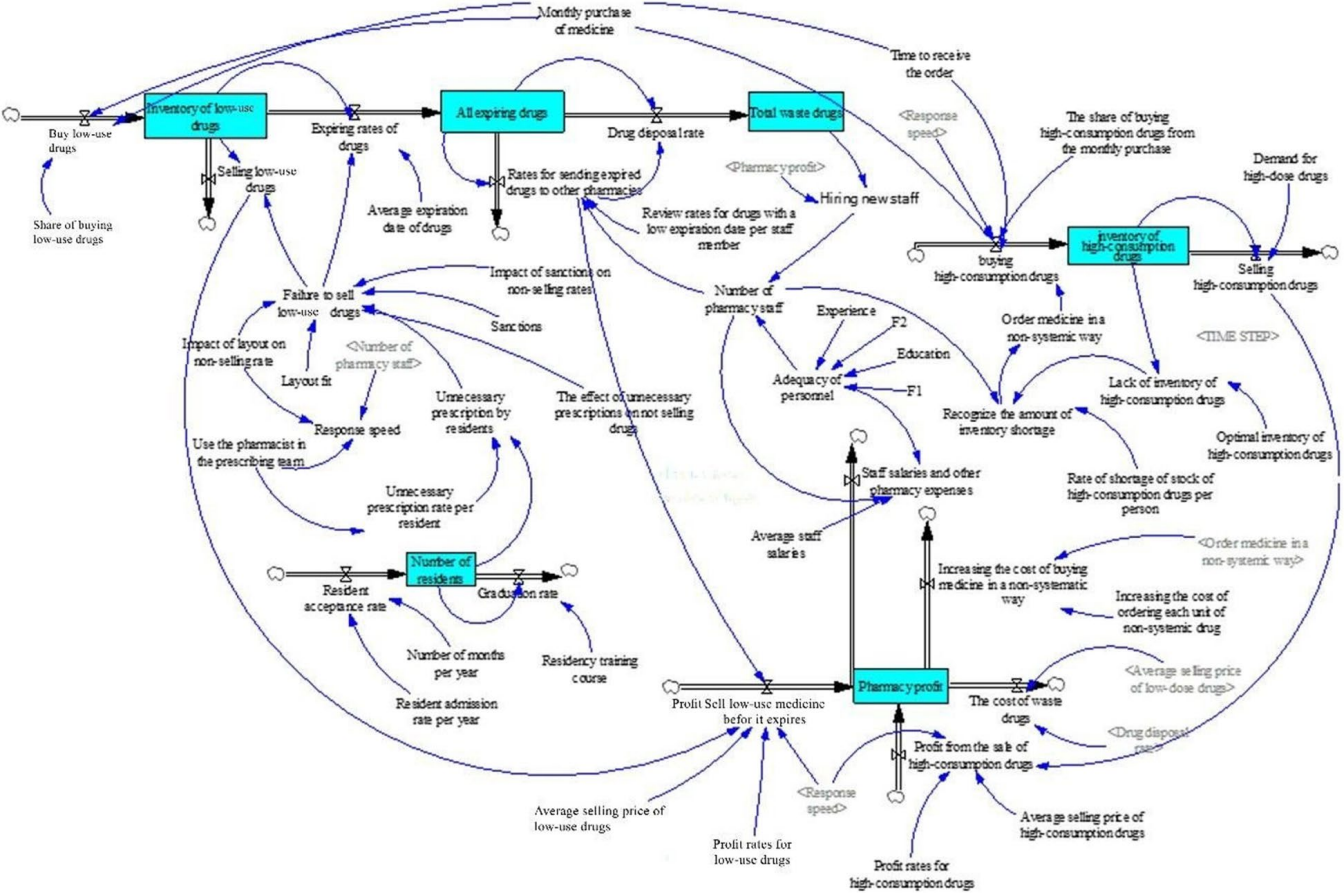
Stock and flow diagram of sustainable drug supply chain management in inpatient pharmacies

The construction of a Stock and Flow Diagram for a system involves utilizing various sources of information, including subject literature, expert opinions, and data obtained through sampling over time. These sources contribute to extracting the necessary variables and relationships within the system. Figure 2 demonstrates the incorporation of quantitative formulas, qualitative relations, and numerical functions during the diagram’s construction.

The figure depicts a model with many variables characterized by complex and nonlinear relationships. Given the complexity and nonlinearity, it becomes challenging to model such dynamics using methods other than system dynamics. Figure 2 exemplifies this situation, where Stock variables such as pharmacy profit, number of residents, total expiring medications, total disposed medicine, and inventory of high-consumption medicines are included. The Flow variables associated with these Stocks change based on input and output prices.

For instance, variables like the sales of low-consumption medicine before expiration and the profit from selling high-consumption medicine are considered input variables for the pharmacy profit Stock variable. On the other hand, variables such as the cost of disposed medicine, personnel wages, overhead costs of the pharmacy, and average personnel salaries are regarded as output variables influencing the pharmacy profit Stock variable.

Once the causal relationships within the system have been analyzed and understood, the next step is to test the complete system dynamics model. The testing process should be conducted systematically and purposefully. By conducting careful and deliberate simulation experiments, valuable insights can be gained into the real problem and the functioning of the systems involved in the problem situation. This process aligns with the fundamental objective of system dynamics modeling, which aims to understand the behavior of the actual system.

Testing the system dynamics model involves running various simulation experiments and observing the model’s behavior. These experiments help analyze the system’s dynamics, identify patterns, and assess the model’s performance. A deeper understanding of the system can be obtained by examining the behavior of the Stocks and Flows within the model.

**Fig. 3:**
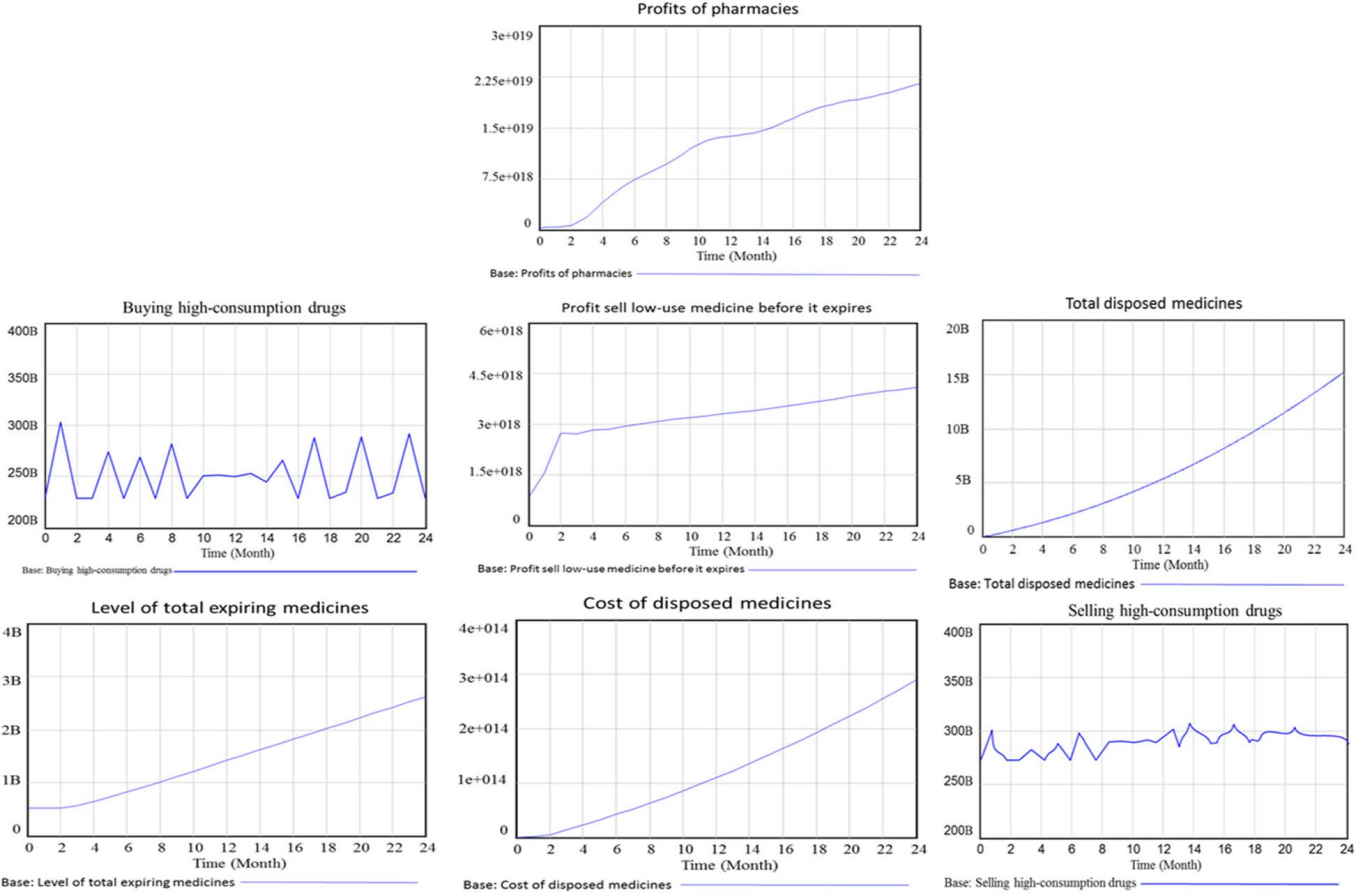
Simulated behaviour of some variables over a period of 24 months

Figure 3 illustrates the behavior of selected simulation factors in the Stock and Flow Diagram. This diagram visually represents the interactions and dynamics among these factors.

#### Variable - total disposed medicines

According to the simulation diagram and the relationships identified, the variables of total expiring medicines, the rate of disposed medicines, and the exchange rate between pharmacies influence one another. Based on this information, it can be inferred that the trend of disposed medicines over the next 24 months will be upward, indicating an increasing wastage rate.

Moreover, the rate of increase in disposed medicines is expected to be even higher during the second 12-month period compared to the first 12 months. This suggests that the growth in the total wastage of medicines will follow an exponential pattern. Consequently, pharmacies will face rising costs and reduced profits due to increased wastage of medicines.

Additionally, the simulation results indicate that the overall trend of expiring medicine will exhibit an upward trajectory over the next 24 months. However, it is noted that the trend remains stable during the initial months, specifically the first two months.

#### Variable - level of total expiring medicines

The delivery rate of expiring medicines to other pharmacies can significantly influence the behavior of the variable related to medicine disposal/expiration. If the delivery rate is low or inefficient, it can contribute to the accumulation of expiring medicines within pharmacies. This, in turn, can lead to a higher rate of medicine disposal or expiration, increasing the total waste of medications.

#### Variable - cost of disposed medicines

As per the flow diagram in the research, the variable of medicine disposal rate, along with the average selling price of low-consumption medicines, influences the behavior of the variable associated with pharmacies’ profits. The disposal rate of medicine refers to the rate at which pharmacies discard or waste medicines.

The flow diagram suggests that this variable directly impacts pharmacies’ profits. When the disposal rate of medicine increases, the costs associated with wasted medicine also increase. These costs can include disposal fees, write-offs, or any other costs incurred due to the disposal of medicines.

The simulation results indicate that the cost trend of disposed medicine is expected to increase over the next 24 months. This suggests a rising trend in the costs associated with wasted medicine. Furthermore, the rate of increase in the second 12 months is higher than in the first 12 months, emphasizing the need for pharmacies to pay close attention to this trend.

#### Variable – sales of low-consumption medicine before expiration

This variable exerts a direct influence on the profitability of pharmacies. Its behavior is shaped by several factors, including response speed (the timely processing and delivery of medicines, as well as providing accurate advice and addressing customer queries), the rate of distributing expiring medicine to other pharmacies, the profit rate associated with low-consumption medicine, and the average selling price of such medicines.

The results indicate that the overall trend of sales for low-consumption medicines before expiration is projected to increase over the next 24 months gradually. However, it is noteworthy that there is a notable surge in sales during the initial two months, followed by a subsequent period of slower growth.

#### Variable – pharmacy profitability

The most crucial variable in the model, directly reflecting a pharmacy’s performance, is its profitability. This variable’s behavior is directly influenced by several factors, including profit derived from the sales of low-consumption medicines before their expiration, profit from the sale of high-consumption medicines, the cost associated with disposed medicines, staff salaries, and other operational costs incurred by the pharmacy.

The results indicate that the overall trend in pharmacy profitability will increase over the next 24 months. Notably, the rate of increase during the first 12 months is higher. This can be attributed to factors such as the rising trend in total wasted medicines and the associated costs of disposing of these medicines.

#### Variable – buying high-consumption drugs

One of the other variables in the model that affects the profitability of pharmacies is “Buying high-consumption drugs.” The behavior of this variable is influenced by the variables of response speed, the share of buying high-consumption drugs from the monthly purchase, time to receive the order, and monthly purchase of medicine. Based on the simulation graph in Fig. 3, the overall trend of “Buying high-consumption drugs” over the next 24 months fluctuates sinusoidally with increases. It decreases proportionally to the volume of orders for this type of drug.

#### Variable – selling high-consumption drugs

Among other variables in the model that impact profitability, the variable of high-consumption drug sales plays a significant role. The variables of inventory of high-consumption drugs and demand for high-consumption drugs influence the behavior of this variable. As depicted in the simulation graph in Fig. 3, the overall trend of high-consumption drug sales over the next 24 months follows a sinusoidal pattern, increasing and decreasing monthly, similar to the purchasing levels.

### Step 4. Validating the model

To ensure the validity of the model’s performance, several tests were conducted.

- Structure Verification Test.

This study presents a sustainable drug supply chain model in hospital pharmacies affiliated with Iran’s Medical Sciences Universities. The model has been developed at a macro level based on a natural system, and it incorporates a comprehensive visual representation of the system and its workflow. The model’s structure is consistent with the existing knowledge of the actual system and aims to simulate the most relevant aspects of the actual system. Expert opinions were also sought to ensure the model’s relevance and alignment with the real-world system.

b) Parameter Verification Test.

Given that the variables employed in the model are based on extensive background research, the parameter values were derived from documentation obtained from 17 hospital pharmacies affiliated with Iran University of Medical Sciences. Additionally, expert interviews were conducted to ensure the accuracy and alignment of these parameter values with real-world conditions. Thus, it can be asserted that the selected parameter values reflect actual values prevailing in the studied context.

c) Boundary – Adequacy (Structure) Test.

**Fig. 4:**
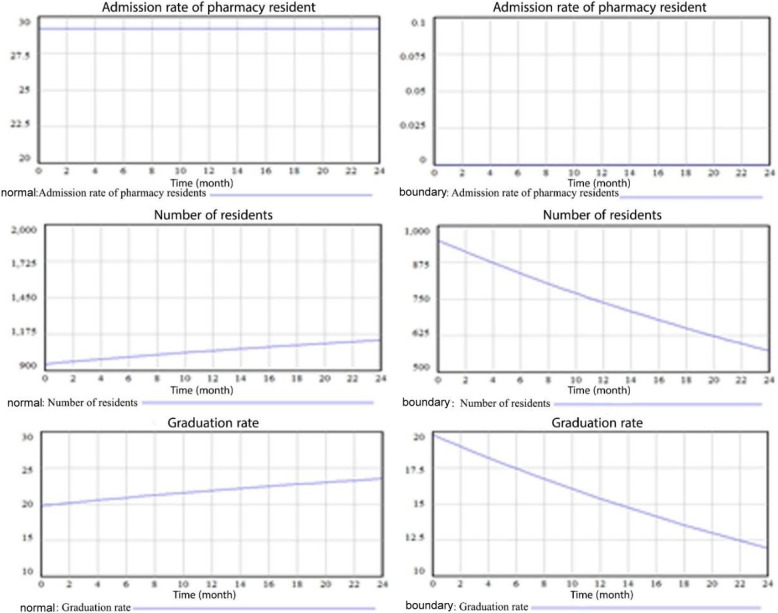
Graph of resident pharmacy rate, number of residents and graduation rate variables in normal and boundary conditions

In order to conduct this test, the behavior of critical variables in the model, namely the resident pharmacy rate, the number of residents, and the graduation rate, was evaluated under the assumption of approaching a minimal value (zero). Figure 4 presents the outcomes obtained from applying these zero-limit conditions. The simulated behavior of the variables demonstrates that they exhibit rational and acceptable patterns. Specifically, when one variable experiences a significant decrease, its dependent variables also decrease as anticipated.

### Step 5. Policy design and evaluation

A vital advantage of the system dynamics approach is its ability to accommodate various scenarios and facilitate result comparisons. Multiple future scenarios can be developed once a reliable model with an enhanced policy structure is established. At this stage, diverse management options can be explored by manipulating systemic and policy parameters. The impact of these variations on the dynamic behavior of the model can be observed, generating a range of future scenarios.

Different decisions can be made within each scenario, considering the specific problem and its associated results. This allows for the evaluation of different policies and their effectiveness. By comparing these scenarios, the relative merits and drawbacks of different policies can be assessed, aiding decision-making processes. This approach provides a valuable tool for exploring alternative pathways and identifying the most favorable strategies for addressing complex problems.

### Scenario 1: increasing the competence of human resources

**Fig. 5:**
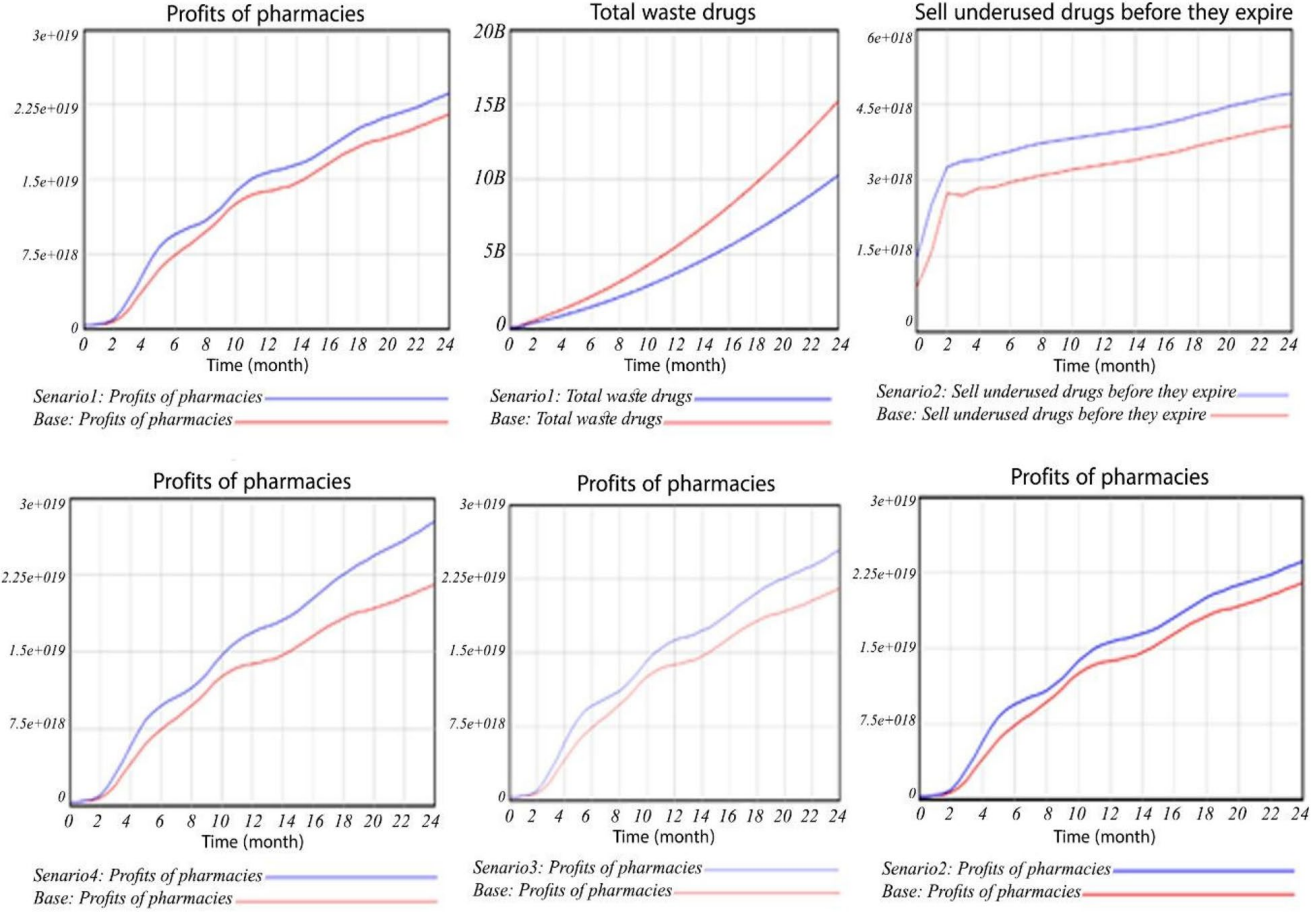
Changes in the main variables of the research under the four research scenarios

In this scenario, the workforce competence variable was doubled, and its impact on the behavior of pharmacy profit can be observed in Fig. 5. The figure demonstrates that with an increase in workforce adequacy (doubled in this case), the profit of pharmacies also exhibits a steady upward trend. This observation suggests that the adequacy of human resources, influenced by factors such as employee training and experience, plays a significant role in determining the profitability of pharmacies.

### Scenario 2: increase the response speed

In this scenario, the response rate is doubled, as reflected in the depicted profit amount. Initially, with the increased response speed in the first few months, the profit amount is lower compared to the baseline case. This could be attributed to reduced accuracy and decreased staff concentration during the adjustment period. However, the profit gradually increases at a steep rate afterward.

Therefore, increasing the response speed by augmenting the number of staff and involving pharmacists in the prescribing team can significantly impact pharmacy profits. Additionally, the results indicate that as the response speed increases (doubled in this scenario), the expiration rate of medicines decreases. This suggests that a faster response rate, influenced by factors such as staffing levels and the involvement of pharmacists in the prescribing process, can effectively reduce the rate of medicine expiration.

### Scenario 3: using a pharmacist in the prescribing team

In this scenario, the number of pharmacists involved in the prescribing team was reduced by half. The outcome of this reduction is a decrease in profit. Hence, it can be concluded that utilizing a pharmacist team in the prescription process significantly impacts the profitability of pharmacies. The involvement of pharmacists in the prescribing team enhances response speed, enabling the sale of low-consumption medicines before their expiration dates. Consequently, this contributes to an increase in pharmacy profits.

### Scenario 4: simultaneous increase of human resource competence, response speed and use of pharmacist in the prescription team

Figure 5 presents the outcomes of augmenting workforce adequacy, response speed, and the integration of pharmacists in the prescribing team, as well as the combined implementation of all three scenarios on pharmacy profits. The figure demonstrates a consistent upward trend in profits as workforce adequacy, response speed, and pharmacist involvement in the prescribing team increase.

## Discussion

This study presents a dynamic and sustainable supply chain management model designed explicitly for pharmacies in educational hospitals. The initial scenario analysis yielded results indicating that an increase in workforce adequacy is associated with enhanced profitability for pharmacies and reduced waste. Previous research has also highlighted the crucial role of a skilled and trained workforce in successfully attaining pharmacy objectives [16, 19]. Insufficient human resources can undermine service quality provided to patients. Consequently, an accurate estimation of the required staff (including pharmacists, experts, technicians, and warehouse personnel) in alignment with pharmacy activities is strongly recommended. Additionally, efforts should be made to enhance the knowledge level of pharmacy staff by utilizing educational books and magazines or organizing training sessions.

Moreover, Heydari et al. emphasized the importance of training hospital staff to address potential disruptions in the supply chain. It is further suggested that additional training courses be provided to enhance the workforce’s expertise [17, 18].

A practical proposal to enhance workforce adequacy is designing and developing a system for managing and evaluating human resources performance in pharmacies. This system would enable the effective monitoring and assessment of employee performance, aiding in identifying areas for improvement and optimizing resource allocation. Criteria such as experience, education (specifically related expertise), communication skills, and decision-making abilities should be considered in the recruitment and employment processes to ensure the selection of competent individuals. By implementing such a system, pharmacies can improve the overall quality of their workforce and enhance operational efficiency.

The second scenario analysis revealed that an increase in response speed initially leads to lower unit profitability in the first few months, followed by a significant subsequent increase. Enhancing response speed by augmenting staff numbers and involving pharmacists in the prescribing team can positively impact pharmacy profits. These findings were not observed in similar studies. Consequently, it is recommended that customer response practices be implemented utilizing internet-based tools, telephony systems, and other information and communication technology tools to expedite addressing customer needs and requests. Moreover, reducing clerical and administrative burdens on pharmacists, enabling them to focus on their primary responsibilities, is advocated. Employing modern and efficient procurement methods from drug centers and supplier companies is suggested to prevent shortages of high-consumption medicines. Additionally, continuous efforts by the Food and Drug Administration to expedite exercises and eliminate medication and supply shortages are recommended as executive measures for pharmacies to enhance their responsiveness.

Furthermore, a separate study highlighted the implementation of strategic stocking practices and the utilization of multiple supply sources, especially for imported medicines, as potential policies for sustainable chain management in Iranian pharmacies. Leveraging the capacities of cyberspace and social networks to respond to patients promptly is also proposed as a means to enhance response speed and ultimately increase patient satisfaction.

The implementation of the third scenario demonstrated the significant impact of incorporating pharmacists in the prescribing team on pharmacy profits. These findings were not observed in similar studies. Therefore, it is advisable to establish guidelines and regulations mandating the involvement of pharmacists in the prescription dispensing process. Article 2 of the Law on Medical, Pharmaceutical, Food, and Beverage Regulations designates the pharmacy’s technical manager responsible for medication dispensation. However, this law has frequently been violated, resulting in the absence of pharmacists from pharmacies. This situation risks public health and tarnishes the country’s scientific reputation. To address this issue, it is recommended that Iran’s pharmacy system be upgraded through collaboration among various stakeholders, the development and implementation of evidence-based national guidelines, and the enhancement of the FDA’s capacity to regulate the pharmaceutical market. Pharmacists should also be readily available as reliable consultants throughout the day, providing standard treatment recommendations and medication advice.

In addition, it is essential to acknowledge the limitations of this research, one of which is the lack of accurate statistical data in pharmacies. This limitation highlights the need for future studies to address this issue. We recommend that researchers conduct studies focusing on the Pathology of Information Systems and knowledge management in pharmacies or the Design of Pharmacy Information Systems. Exploring these topics can provide valuable insights into improving data collection and management in pharmacy settings. The lack of accurate statistical data poses a challenge for managers and decision-makers in the country’s medical system, and addressing this issue is crucial for evidence-based decision-making and effective resource allocation.

Furthermore, future researchers can consider incorporating additional factors into the research model to enhance its comprehensiveness and accuracy. Factors such as electronic medical records, the implementation of clinical pharmacy practices, medication reconciliation processes, considerations of drug formulations or preferences between generic and trade medications, and the verification process of pharmacy orders and prescriptions can be included. By expanding the model’s scope to include these elements, a more comprehensive understanding of the factors influencing pharmacy profitability and performance can be achieved.

## Conclusion

The scenarios of “increasing the adequacy of human resources,” “increasing the response speed,” and “using the pharmacist in the drug prescribing team” have been identified as having a substantial impact on increasing pharmacy profitability and reducing medication waste. These findings highlight the importance of optimizing workforce capacity, improving response times, and leveraging pharmacists’ expertise in prescribing to enhance overall pharmacy performance. Optimizing human resources, improving response times, and leveraging pharmacists’ expertise are vital strategies for enhancing pharmacy profitability and reducing medication waste. These improvements benefit the pharmacy’s bottom line and contribute to better patient care and satisfaction.

## Data Availability

The datasets used and/or analysed during the current study are available from the corresponding author on reasonable request.
